# Computational Approaches for Pathway‐Centric Analysis of Protein Post‐Translational Modifications

**DOI:** 10.1002/pmic.70055

**Published:** 2025-10-18

**Authors:** Julian Müller, Bernhard Kuster, Matthew The

**Affiliations:** ^1^ Proteomics and Bioanalytics School of Life Sciences Technical University of Munich Freising Germany; ^2^ German Cancer Consortium (DKTK) Partner Site Munich, a partnership between DKFZ and Technical University of Munich (TUM) Munich Germany

**Keywords:** computational biology, enrichment analysis, pathways, post‐translational modifications

## Abstract

Protein function is dynamically modulated by post‐translational modifications (PTMs). Many different types of PTMs can nowadays be identified and quantified at a large scale using mass spectrometry. It is well known that many PTMs have an effect on protein function and cellular processes, and they should be studied not in isolation, but in the holistic context of cellular pathways. This is increasingly facilitated by a wide variety of computational efforts. This review aims to give a systematic overview of tools for pathway‐centric analysis of PTM data and critically evaluate the state of play in this research field. Starting from databases that make up the foundational prior knowledge, we follow typical steps that an analytical workflow might contain, including pathway enrichment analysis, algorithms for pathway reconstruction, and the integration and visualization of results. We then reflect on common limitations of all existing tools and give our opinion on future directions that we think are currently most desirable.

**Abbreviations**
API: application programming interface
FCS: functional class scoring
GSEA: gene set enrichment analysis
ORA: overrepresentation analysis
PCST: prize‐collecting Steiner tree
PKN: prior knowledge network
PPI: protein–protein interaction
PSP: PhosphoSitePlus
PT: pathway topology
RWR: random walk with restart
SPIA: signaling pathway impact analysis
ssGSEA: single sample gene set enrichment analysis
TPS: temporal pathway synthesizer

## 1 Introduction

The study of post‐translational modifications (PTMs) has evolved from a specialized research niche into a central pillar of molecular biology. This shift reflects the growing understanding that protein function is not solely determined by protein primary structure or expression levels, but strongly modulated by dynamic biochemical modifications [1]. PTMs act as a molecular language through which cells encode responses to environmental cues, regulate signaling cascades, and fine‐tune metabolic processes. This additional regulatory layer enables rapid cellular adaptation without the energy costs of de novo protein synthesis [2]. PTMs operate within complex and tightly regulated networks, governed by writer and eraser enzymes. For example, phosphorylation is controlled by kinases and phosphatases; acetylation by lysine acetyltransferases and deacetylases; and ubiquitination by E3 ligases and deubiquitinases. These enzymes form regulatory circuits that facilitate information transfer, signal integration, and crosstalk, and a change in enzymatic activity can have effects all across a cell. The study of such networks has been one of the main concerns of systems biologists for decades, and it has led to the creation of many experimental protocols and, subsequently, a number of databases and software. Technological advances have by now enabled high‐throughput measurements of various PTM types: for instance, phosphoproteomic platforms such as µPhos can quantify over 17,000 phosphosites per sample [3], and ubiquitomics workflows using K‐ε‐GG antibodies can detect several thousand ubiquitination sites [4]. Meanwhile, integrating pathway information into the analysis of PTM data is not straightforward, and the number of available options can be overwhelming.

**FIGURE 1:**
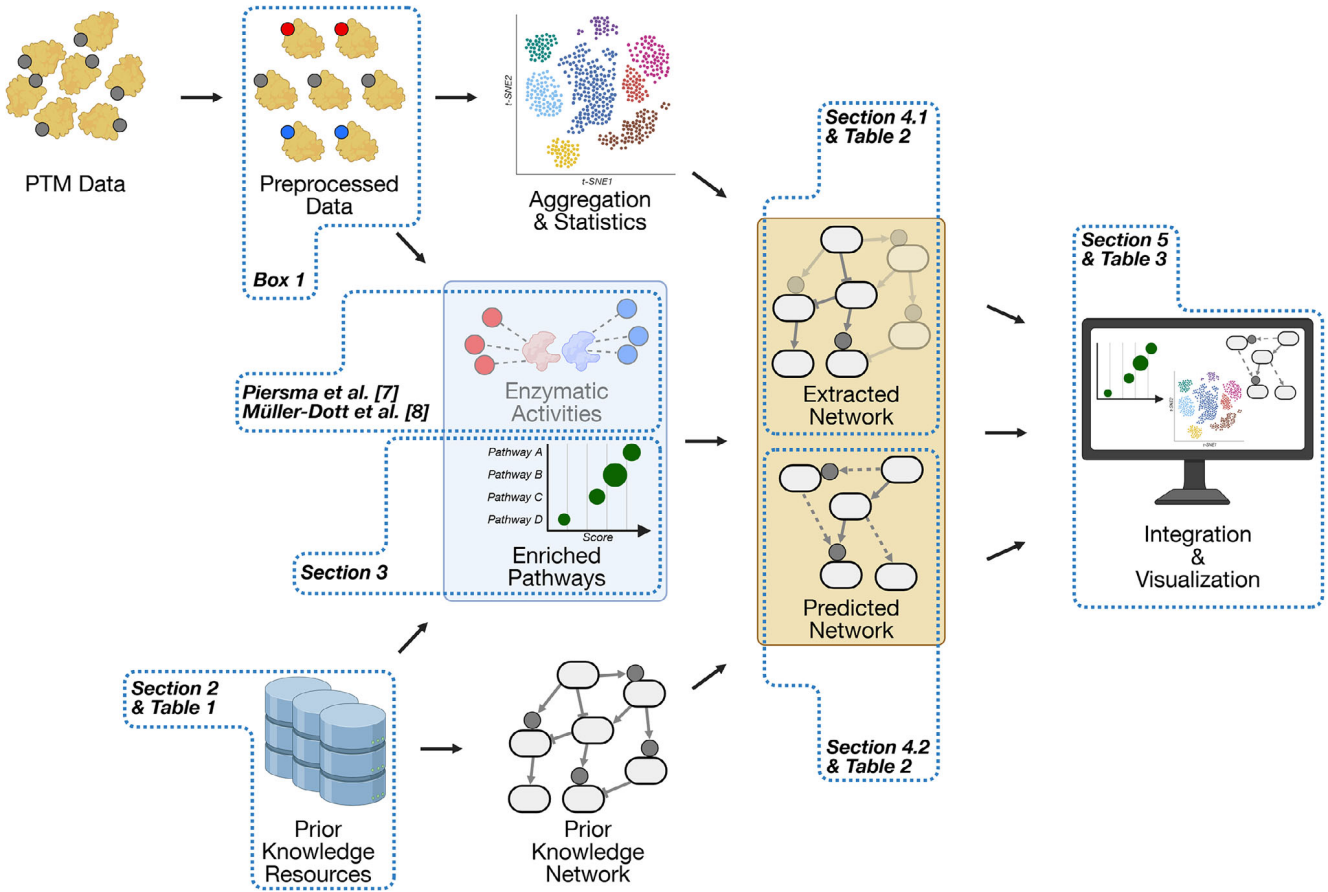
Schematic workflow for a pathway‐centric PTM data analysis. The prediction of enzymatic activities is closely related to pathway enrichment and has recently been discussed by multiple other reviews (see main text).

In this review, we provide an overview of the computational tools available for pathway‐centric PTM analysis. We constructed what we consider a reasonable sequence of analysis steps (Figure 1), and matched representative methods and resources to each of the steps. Following this exemplary workflow, we begin with a comparison of databases that are relevant to the topic. While some are dedicated exclusively to information on PTMs, others also combine it to various extents with pathway knowledge. Moreover, some databases only exist as static tables, whereas others offer graphical tools to gain deep insights into prior knowledge. Then, we move to methods that combine this knowledge with experimental PTM data to identify enriched pathways. For gene‐centric analyses, this has been a well‐established technique for some time, but not so much on the PTM level. We discuss what methods exist and how transferable the gene‐centric algorithms are. Since every enrichment analysis only returns a list of canonical pathways or biological functions that are limited to the annotations in the employed database, many users will want to zoom in more deeply or find pathway architectures that are specific to the context of their model system and experimental condition. Therefore, the subsequent section of our review is dedicated to data‐driven pathway reconstruction methods. We distinguish between “network extraction” tools that leverage prior knowledge networks (PKNs) to extract the interactions that are most relevant to an input dataset, and “network prediction” tools that predict novel links or pathways ab initio. Finally, we take a look at platforms that integrate and visualize the results from other tools. These are especially attractive for users with a non‐bioinformatics background, as they often include graphical implementations of enrichment or reconstruction algorithms that would otherwise require programming skills to various extents.

Here, we focus specifically on representative tools that assist in the interpretation of PTM data in the context of pathways. For closely related topics, that is, PTM data preprocessing, enzyme–substrate prediction, enzyme activity inference, and multi‐omics integration, we refer to recent high‐quality reviews described below.

Savage and Zhang, as well as Xiao et al., reviewed methods for phosphoproteomics data analysis strategies. While the former publication puts the focus on databases and tools available at the time of writing (2020), the latter includes a detailed description of preprocessing strategies [5, 6]. To complement this, we decided to include a list of what we consider common pitfalls in PTM data preprocessing in this publication (see Box 1). A topic that is closely related to pathway enrichment is the prediction of enzymatic (mostly kinase) activity. We decided to focus only on pathways in this review, and recommend recent publications by Piersma et al., who compared several popular kinase activity inference tools [7], and Müller‐Dott et al., who comprehensively benchmarked not only methods, but also different kinase–substrate libraries [8]. Lastly, Garrido‐Rodriguez et al. recently published a review on network reconstruction using various omics, including phosphoproteomics [9], while Franciosa et al. wrote a review focused on the elucidation of kinase signaling networks [10].

**Box 1. Considerations for PTM data preprocessing**

When preparing a large‐scale PTM dataset before downstream analysis, one needs to handle various types of ambiguity. In our opinion, there is usually no right or wrong, but especially when comparing different datasets, it is important that these ambiguities are all resolved in the same manner.

- The measurements are made on the level of peptides, but most analysis tools require site‐level data; therefore, one has to handle peptides that contain multiple PTMs. Options include using the measurement multiple times, that is, assigning each of the sites the quantitative value from the peptide (which could, however, introduce a skew in the distribution of the data), removing the peptide altogether (only allowing unambiguous PTMs for the analysis), or using advanced statistics like the linear mixed model approach implemented by msqrob2PTM [11]. Our preferred option is reusing the quantitative value, with the caveat that one avoids counting the same measurement multiple times in downstream analyses, such as enrichment or enzyme activity analysis.
- A second issue is PTMs for which multiple measurements exist, which can be the result of a missed cleavage or, again, the peptide being measured with and without additional PTMs. This can again be resolved by approaches such as taking an aggregate, such as the mean, sum, minimum, or maximum value across all observations for the PTM, or by the removal of any ambiguous PTM, or by linear mixed models. While this potentially reduces the size of the dataset, it avoids analytical pitfalls such as the following: If a PTM is measured only in combination with another PTM, and the occupancy of this multi‐PTM decreases between conditions, the reason could be that one of the singly modified (but not observed) variants actually increased in occupancy, leading to an apparent decrease in the multi‐PTM peptidoform. In this case, it would be wrong to assign the decrease to both sites. We, therefore, recommend removing such ambiguous cases from the analysis.
- Third, a peptide can often be attributed to multiple proteins, typically because the protein has multiple isoforms. This can be resolved by selecting only canonical isoforms, only the proteins with the most literature evidence (or annotation level in UniProt), or simply by including all possible annotations in the analysis. Depending on the type of analysis, the last option can introduce an unwanted bias in the data (overrepresentation of sites simply because their proteins have many isoforms). For simplicity, we, therefore, recommend only attributing the PTM to the canonical isoform.
- Last, PTMs are often ambiguously localized within the peptide sequence due to the absence of peptidoform‐typic ions in the fragment mass spectra. Note that this is different from (A), where instead, multiple modifications are present at the same time in a single peptide. Search engines usually output probability scores for the positions of modifications, but these have been shown to be of limited confidence [12, 13]. To some extent, this can be improved using additional software such as PyAscore [14], but some uncertainty often remains. Users can either retain only the sites that exceed a certain probability threshold or keep all sites and attach the probability to the quantitative value. The former is considered best practice to ensure control over the false localization rate [12, 13], but in our experience, the latter works well for downstream analyses where multiple PTMs are aggregated, for example, by enzyme or protein.

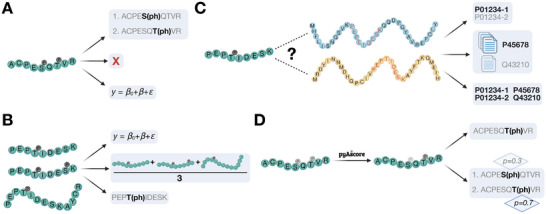

Common pitfalls when preprocessing large‐scale PTM datasets. A. Options for processing peptides that contain multiple modified sites. B. Options for processing PTMs which are measured in multiple peptidoforms. C. Options for resolving the ambiguous assignment of PTMs to proteins. D. Options for resolving localization ambiguity of PTMs within a peptide.

We conclude our review with a critical perspective on the field's current state and propose future directions for methodological and conceptual development.

## 2 PTM and Pathway Databases

A vast number of databases that contain collective knowledge and/or experimental data on PTMs and biological pathways have been established. While most of these resources are designed with a primary focus—either on PTMs or on pathways—some offer functionality that supports analysis from the complementary perspective. We examined several representative examples, the main characteristics of which are compared in Table 1. In general, we found that there is a bias towards phosphorylation. This is in part, justified, since it is considered to be the most important PTM regarding cellular signaling [15] and may also be more frequently studied because it is comparatively simple to enrich and therefore easier to detect in high‐throughput experiments.

**TABLE 1:** Public databases with a focus on PTMs and/or pathways.

Database name	URL	PTMs included	Species covered	Size	Last update (as of June 2025)	Usability features	Site function information	Enzyme–substrate relationships	License
PhosphoSitePlus [16]	https://phosphosite.org	Phosphorylation, ubiquitination, acetylation, methylation, O‐glycosylation	Human, mouse, rat (sparse data on 24 other species)	∼380,000 sites, ∼24,000 kinase–substrate relationships	May 2025	Download option after registration and licensing	Yes	Yes	Web use free; license required for download (free for academic use)
iPTMNet [17]	https://research.bioinformatics.udel.edu/iptmnet	8 types, including phosphorylation, acetylation, ubiquitination	15 metazoa, fungi, and plants	∼830,000 sites	January 2024	Tutorial, API, download	No	Yes	CC BY‐NC‐SA 4.0
EPSD [18]	https://epsd.biocuckoo.cn	Phosphorylation	222 eukaryotic species (incl. human, mouse, rat, fly)	∼2,700,000 sites	June 2025	User guide, download	Yes	Yes	Free
iKiP‐DB [19]	https://pubs.acs.org/doi/abs/10.1021/acs.jproteome.2c00198 (only available as supplementary table)	Phosphorylation	Human	6382 unique phosphorylation sites, 313 signatures (mean size: 88)	2022	Only available as supplementary table	No	Yes (in vitro from Sugiyama et al.)	Free
The Kinase Library [20, 21]	https://kinase‐library.mit.edu	Phosphorylation	Human	Binding preferences for 311 Ser/Thr Kinases and 93 Tyr Kinases	April 2025	Web tools for scoring and enrichment are available	No	Yes (in vitro from Johnson et al. and Yaron‐Barir et al.)	CC BY‐NC‐SA 3.0
PTMsigDB [22]	https://github.com/broadinstitute/ssGSEA2.0	11 types, >99% of entries are phosphorylation	Human, mouse, rat	Human: 18,930 unique phosphorylation sites, 955 signatures (mean size: 70)	2023	Only available on GitHub	Yes	Yes (from PSP)	CC BY 4.0
ActiveDriverDB [23]	https://activedriverdb.org	7 types	Human	∼261,000 PTM sites, 18,500 pathways	2021	Tutorial, API, PTM‐affecting mutations can be downloaded	Disease annotations	Yes	LGPL 2.1
dbPTM [24]	https://biomics.lab.nycu.edu.tw/dbPTM	76 types	50 organisms	2.84 million sites	2025	Tutorial available, download available	Yes	Yes	Free
FAT‐PTM [25]	https://fat‐ptm.tinnguyen‐lab.com	8 types	*A. thaliana*	49,000 sites, 600 pathways	2019	Help page, pathway editor available after registration	No	No	Free
SIGNOR [26]	https://signor.uniroma2.it	17 types	Human (mouse & rat via orthologues)	39,000+ interactions	April 2025	Tutorial, documentation, API, download available	Yes	Yes	CC BY 4.0
WikiPathways [27]	http://wikipathways.org	Not reported	27 organisms	2006 pathways	June 2025, Updated almost daily	Tutorial, API, download of pathway diagrams	No	No	CC0 1.0
BioCyc [28]	https://biocyc.org	Not reported	40 eukaryota, 19,448 bacteria, 467 archaea	2500 pathways (across organisms)	April 2025	Tutorial videos, download, API	No	No	Paid license required; discount for academic users
KEGG [29]	https://www.kegg.jp	Phosphorylation, methylation, glycosylation, ubiquitination	1184 eukaryotes, 9279 bacteria, 449 archaea	6782 pathways	April 2025	API, individual pathway diagrams can be downloaded	No	Yes (on protein‐level)	Commercial use requires paid license
Reactome [30]	https://reactome.org	Not reported	*H. sapiens*	2769 pathways	March 2025	Chatbot, documentation, tutorial, API, download	Yes	Yes	Pathway diagrams: CC BY 4.0 Data: CC0 1.0
OmniPath [31]	https://omnipathdb.org	20 types	Human (mouse & rat via orthologues)	43,000+ enzyme‐PTM interactions	May 2023	API + Python/R wrapper	Yes	Yes	MIT
KiNet [32]	https://kinet.kinametrix.com	Phosphorylation	*H. sapiens*	∼11,000 sites ∼202 pathways	July 2024	Download available	No	Yes	Free

One of the largest and most popular resources is PhosphoSitePlus (PSP), maintained by Cell Signaling Technology [16, 33]. Despite its name, it is not limited to phosphorylation, but also incorporates experimental data from four other PTMs. It is frequently updated with both high‐ and low‐throughput data (currently from more than 31,600 publications). This is complemented by almost 24,000 kinase–substrate relationships, allowing users to connect phosphosites to up‐ and downstream regulators. Several other databases reuse the data from PSP. Notable examples include iPTMnet [17] and EPSD [34]. The former complements the information from PSP and other primary resources with knowledge extracted from the literature via text mining. The authors of the latter manually curated a subset of PSP and more than 700 phosphoproteomic studies and combined it with sources of related information (such as disease associations or drug‐target relations). In early 2025, a major update to this database was published [18]. An effort that is independent of PSP is iKiP‐DB [19]. It is a kinase–substrate library compiled from in vitro kinase assays by the Ishihama lab [35]. This resource has the advantage of avoiding a potential study bias, since every kinase is treated the same in the underlying experiment. The in vitro setting, however, only assesses the sequence specificity of each kinase; it does not reveal whether a kinase and its putative substrate would actually ever encounter one another in a live cell. Also, the preparation process of the assay required a denaturation step, potentially resulting in binding at regions that are not usually exposed to enzymes. A similar, more recent effort is the Kinase Library, which employed a synthetic peptide library to profile the substrate specificity of 303 human serine/threonine kinases [20] and all human tyrosine kinases [21]. The resulting dataset can be explored online, and the most likely upstream kinases of any phosphorylation site can be predicted using position‐specific scoring matrices. The authors recommend not using the resource for individual kinase–substrate predictions, since the use of synthetic peptides limits the confidence in these predictions. Instead, users can upload phosphoproteomic datasets and perform kinase activity enrichment analyses. When used in this higher‐level context, the predicted associations have been shown to be beneficial for data interpretation.

Beyond studying individual kinase–substrate relationships, no high‐level pathway analysis is directly possible with any of the databases listed so far. PTMsigDB, in turn, is a resource that associates phosphorylations not only with kinases, but also with canonical pathways, drug responses, and diseases [22]. It is built from multiple resources, including PSP and iKiP‐DB. The signatures are available in three formats: ±7‐flanking, Uniprot + Position, and PSP site group identifier. Another notable property is that the annotations are bidirectional, a crucial information that is often overlooked when performing analysis. However, its coverage of the proteome is quite low in comparison to its gene‐level counterpart MSigDB [36]. A pan‐PTM database that offers a graphical web frontend is ActiveDriverDB [23]. It is dedicated to the collection of mutations in human genes that have an effect on PTMs. The homepage includes a network view, where for a protein of interest, all known mutations and the affected enzyme–substrate relationships can be visualized. The website allows the upload of custom single‐nucleotide variant data, so users can analyze the impact of their observed mutations on the PTM level. The focus of the database is on phosphorylation, but it also includes, for example, glycosylation or sumoylation. All PTM information is collected from other databases (mostly PSP). There also exist meta‐databases, such as dbPTM [24]. It incorporates more than 40 individual resources, including PSP, ActiveDriverDB, and, for example, BioGRID [37] and EPSD. According to the publication, 98 types of PTMs are covered; however, we were only able to find 76 on the website. As in PSP, individual enzyme–site relationships are available in tabular format, and it has a network visualization component, but only on the level of protein–protein interactions (PPIs). Lastly, there is FAT‐PTM, which is dedicated to *Arabidopsis thaliana* [25]. It is backed by data from the PhosPhAt database [38] and other individual studies, as well as pathway information from the plant metabolic network [39]. Its network visualization component allows zooming in from a high‐level analysis of a canonical metabolic pathway first to the level of protein isoforms and then to PTMs on those proteins. Notably, FAT‐PTM also supports the creation of custom pathway diagrams for registered users. Additional databases exist, which used to be popular in the past, but we decided to exclude them from this review, either because the resource is not online anymore (PHOSIDA [40]), or the data has not been updated in more than 10 years (Phospho.ELM [41], PTMcode [42]).

Regarding databases with a primary focus on pathways, we found SIGNOR to be the one with the most extensive integration of PTM data [26]. It consists of causal relationships that were manually extracted from the literature (13,050 articles as of June 2025), and the authors report that it covers a third of UniProt's human reference proteome. The site can also be searched for mouse and rat proteins (via orthology mapping), and some of the underlying experimental data also stem from other organisms, such as *Cercopithecus aethiops* or *Cricetulus griseus*. One of SIGNOR's unique characteristics is its integrated pathway visualizer, based on the D3.js JavaScript library [43]. Users can search for a protein or a list of proteins to dynamically create interaction networks. The search engine is also able to find indirect connections, using so‐called “bridge” proteins. The visualization indicates where in the cell the proteins are localized (extracellular, membrane, cytosol, nucleus). While not being PTM‐centric, many of the described interactions involve PTMs, and the advanced filtering options (e.g., “show only phosphorylations that down‐regulate the activity of the target”) make this a very useful tool for researchers interested in PTMs. Each reported interaction includes a link to the source publications, often even including a quote of the sentence where the relationship is mentioned. The website also contains more than 80 pre‐drawn diagrams of signaling and metabolic pathways. Other resources in the field do not offer such ways to combine PTM and pathway analysis. For example, the open‐source effort WikiPathways [27] occasionally includes PTMs in its diagrams. The developers recommend including PTM information as “states” of data nodes when designing pathway diagrams [44], but this is only done for a few pathways, and no systematic analysis of PTM networks is directly possible. The same holds true for the proprietary databases BioCyc [28] and KEGG [29]. Reactome, a database whose functional units are reactions, includes many enzyme–substrate reactions but does not offer dedicated analytics for them [30]. The meta‐database OmniPath has some functionality regarding PTM‐pathway analysis [31]. Two of its five resource types are PPI networks (assembled from, e.g., KEGG and SIGNOR) and enzyme‐PTM relationships (assembled from 11 databases, including PSP and dbPTM). However, interaction with OmniPath is only possible via a web service, namely a RESTful application programming interface (API) that can either be queried directly or by means of a Python and an R package. The latter also offers analytical functionality beyond simple data retrieval, such as plotting PTM networks. While this opens the door to automating analyses and integrating OmniPath into other software, it also constitutes a hurdle for non‐bioinformatics users. Contrarily, the KiNet platform [32] was not developed for programmatic access, but for visual integration of prior knowledge on human kinase–substrate annotations from PSP, iPTMNet, and EPSD. Users of KiNet can query proteins of interest and create dynamic graphs of their known KSRs, coloring kinases by their group according to the widely accepted definition by Manning and colleagues [45]. Links to primary sources of knowledge from the literature are provided as well. The authors also integrated information from KEGG: All kinase–substrate relationships from a KEGG pathway can be shown together in a graph to get a high‐level overview of a signaling cascade. Similarly, knowledge on domains from InterPro [46] was integrated: users can select a domain to show the KSR network of all proteins that share this domain.

## 3 PTM‐Centric Pathway Enrichment Analysis

Enrichment analysis is a common tool in molecular life sciences that has been applied since the early 2000s [47]. It reduces the complexity of such data and thus facilitates its interpretation. The earliest incarnations of such methods are overrepresentation analyses (ORA). Traditionally, in an ORA, the set of genes that were deemed differentially expressed between two conditions in an experiment is compared to a set of genes that is collectively associated with a functional annotation (e.g., a certain pathway). Using statistical tests such as Fisher's exact test or the Chi‐square test, it can be calculated if the overlap between the two sets is larger than can be expected by chance. These types of analyses are also referred to as 2 × 2 table methods due to the use of contingency tables to compare the set sizes [48]. Next, there are functional class scoring (FCS) methods (such as Gene Set Enrichment Analysis [GSEA])^1^ In contrast to the ORA methods, these make use of the whole list of measured genes, ranked by quantitative value. FCS methods then estimate if annotation sets are overrepresented at the top or the bottom of the ranked list (using, for instance, the Kolmogorov–Smirnov test or the Wilcoxon rank sum test). Third, pathway topology (PT) methods also consider the interactions between genes or gene products within a pathway, based on the topologies given by pathway databases [49, 50, 51].

The essential prerequisite to perform any such analysis is a functional annotation of the measured data points, that is, genes, proteins, or PTM sites. These annotations exist and are well established for gene‐ and protein‐level data, but they are not very comprehensive to date on the PTM level. As discussed in the previous section, the only database that currently maps PTMs to pathways is PTMSigDB. Therefore, a common way to perform such an analysis on PTM data is to collapse the data to the gene (product) level, as done, for example, in [53, 54, 55], even though it is generally agreed upon that this practice is flawed. The relative abundance of a protein and the relative occupancy of a PTM site on that protein are generally not correlated. Also, different PTMs on the same protein can be the result of interactions with different enzymes, and they can have different consequences for their host proteins. This is exemplified by RAF1, which can be phosphorylated at S338 to stimulate its catalytic activity [56], whereas phosphorylation at S259 leads to attenuation of its activity [57]. Hence, in this section we give an overview of options to perform actual PTM‐centric enrichments.

### 3.1 ORA

One could theoretically use PTMSigDB or the annotated regulatory sites from PSP to perform an ORA with a PTM dataset. We recommend doing FCS, if the data allows it and only resorting to ORA when dealing with non‐quantitative data (for example, when the data has already been processed in such a way that only regulatory classes (up/down/not regulated) remain). Beyond that, ORA discards any further information that would differentiate the data points. In case of pursuing this strategy, one needs to carefully choose the statistical background (i.e., the bottom right cell in the 2 × 2 table) in order to guarantee that the hypothesis test remains fair. This is already crucial when working with gene‐level data, where instead of using the whole reference genome as background, one should only use those genes that can be measured for the model system of interest (either because of technical limitations or due to the genes not being present in the studied individual). Owing to the transience of most PTMs, selecting an appropriate background is an even bigger challenge for this type of data. For example, all phosphosites observed in a single experiment on the model system of interest are unlikely to cover the entire measurable phosphoproteome of that system, since not all phosphosites were occupied at the time the measurement was taken.

### 3.2 FCS

**FIGURE 2:**
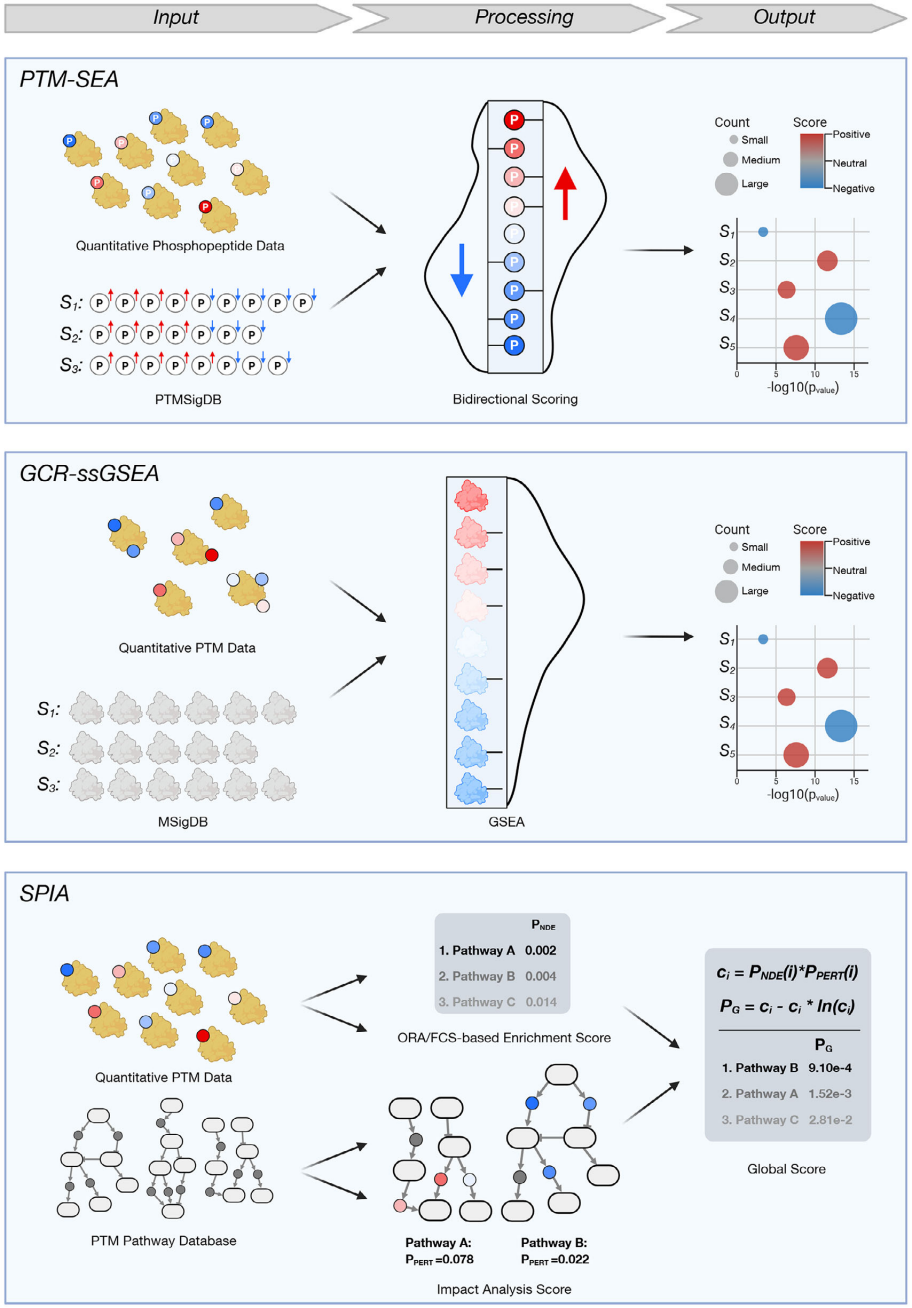
Tools for Pathway Enrichment. Top: PTM‐SEA combines quantitative phosphoproteomic data with a database of directional signatures. A bidirectional and single‐sample variant of GSEA (ssGSEA) is used to calculate a score and *p* value for each signature. Middle: In gene‐centric redundant (GCR) ssGSEA, quantitative PTM data are collapsed to protein level and scored against signatures in the MSigDB database. Genes with multiple PTMs are counted multiple times. Bottom: SPIA is a topological enrichment algorithm designed for gene‐level data. Shown here is a concept of how it could be applied to PTM data. SPIA calculates an enrichment score *P_NDE_* using an overrepresentation analysis (ORA) or functional class scoring (FCS) algorithm and an impact analysis score *P_PERT_* that assesses how the regulated pathway members affect the activity of the whole pathway, based on their position. The two scores are then combined into a global score *P_G_*.

Krug and colleagues developed two FCS methods for PTM research [22]. One, PTM‐SEA, is truly PTM‐centric (Figure 2, Top Panel). It uses the PTMSigDB database (which was created specifically for this purpose) and a modification of the original GSEA algorithm, termed single sample GSEA (ssGSEA). Notably, PTM‐SEA calculates two separate enrichment scores for up‐ and downregulation (which is possible because each signature contains sites associated with both activation and attenuation of the associated biological term—the signatures are bidirectional). For both directions, the data points are sorted by magnitude, and for each site, it is counted how many other sites have a value that is lower or equally large. The sites that belong to a signature are compared to all other sites to determine the score. A signature will receive a high score if its sites are not distributed randomly across the list but instead accumulate at the top or bottom. Scores are normalized, and significance is estimated using permutations. For situations where gene‐centric analysis is preferred (when wishing for higher coverage or dealing with a PTM that is not phosphorylation), the authors showed that it is beneficial to count genes multiple times if they have multiple regulated PTMs, a method they termed gene‐centric redundant ssGSEA (Figure 2, Middle Panel). Both strategies are commonly accepted and regularly used by other groups in the field [58, 59, 60].

### 3.3 PT Methods

While FCS methods are usually preferable to ORA methods (if quantitative data is available), they still essentially treat pathways as “bags of genes.” Both ignore the position of each member within a pathway, which often holds crucial information for the relevance of each component. In contrast, PT methods combine the quantitative data with both functional annotations and the topology of prior‐knowledge interaction networks. They usually require more expertise and are more computationally demanding than ORA or FCS and are carried out less frequently [61].

We are not aware of any PT method that was designed for PTM data analysis, but we believe it would be worthwhile to explore this option. A popular PT method for gene and protein‐level information is signal pathway impact analysis (SPIA) [62]. Ren and colleagues applied SPIA to phosphoproteomics data by using phosphorylated proteins as input (taking the sum over all fold changes of the phosphosites on each protein) and concluded that there is merit in this strategy [54]. SPIA weighs pathway members more heavily if they have a larger influence on the pathway than others (e.g., a node with many outgoing edges receives a larger weight than a leaf node) and combines this with information about differential regulation. We are not aware of a study in which SPIA has been combined with actual PTM networks, but we believe this could be of benefit (the concept of how this could be done is illustrated in Figure 2, Bottom Panel). Similar adaptations could be conceived for methods such as “topology‐based pathway enrichment analysis” (TPEA) by Yang and colleagues, in which the importance of each node in a pathway is estimated based on the node's degree and upstream/downstream position [63].

## 4 Pathway Reconstruction

All the efforts described so far are similar in the sense that they return to the user a scored/ranked list of functional annotations, for instance, canonical pathways or putative dysregulated enzymes. Beyond this, methods exist that return networks that attempt to explain as much of the experimental data as possible. This is an attractive concept because canonical pathways are aggregated over a large biological space (e.g., cell type, cell status, environmental factors, sometimes even organism) and often fail to capture individual situations where all these parameters are fixed. In contrast, pathway reconstruction methods operate in a data‐driven fashion to determine a likely series of protein‐protein or protein‐peptide interactions, the latter being particularly attractive for PTMs. Most of the methods we describe here begin with a PKN of interactions. The objective of these methods is to remove false positive edges and to trim down the graph to a subnetwork of maximal relevance for the input dataset. Since no new predictions can be made, it has to be assumed that the PKN already contains all true positives, that is, that it has a sensitivity of 100%, and that the true underlying pathway(s) can simply be “extracted” from the PKN. We term such methods *extraction methods* to distinguish them from tools that add novel links between interactors (proteins or PTM sites), using prior knowledge to varying extents (we call these *prediction methods*). To assess how well each tool is established in the field, we counted the number of citations, excluding unreviewed preprints and publications that were authored by a first or corresponding author of the respective tool (see Table 2).

**TABLE 2:** PTM pathway reconstruction tools.

Name	URL	Uses prior knowledge?	Directed networks?	GUI available?	Documentation/Tutorial?	Released In	Citations (excluding self‐citations^a^)	License
*Pathway extraction*								
Tuncbag et al. (2013) [64]	https://fraenkel‐nsf.csbi.mit.edu/omicsintegrator/ https://github.com/fraenkel‐lab/OmicsIntegrator2	Yes	Yes	Web‐Based	Yes	2013	73	BSD 3‐Clause
PHONEMeS [65]	https://github.com/saezlab/PHONEMeS	Yes	Yes	No	Yes	2021	13	GPL 3.0
Temporal pathway synthesizer [66]	https://github.com/koksal/tps	Yes	Yes	No	Yes	2018	20	MIT
CausalPath [67]	https://causalpath.cs.umb.edu https://github.com/PathwayAndDataAnalysis/causalpath	Yes	Yes	Desktop and Web‐Based	Yes	2021	43	LGPL 3.0
phuEgo [68]	https://github.com/haoqichen20/phuego	Yes	No	No	Yes	2024	2	GPL 3.0
Ross et al. (2023) (Multi PTM) [69]	https://github.com/m‐grimes/PTMs‐to‐CCCN‐and‐CFN	Yes	No	No	No	2023	9	GPL 3.0
*Pathway prediction*								
Prophetic Granger [70]	https://github.com/decarlin/prophetic‐granger‐causality	Yes	Yes	No	No	2017	8	MIT
DMPA [71]	https://data.mendeley.com/datasets/m3zggn6xx9/draft?a=71c29dac‐714e‐497e‐8109‐5c324ac43ac3	No	No	Desktop‐based	Yes	2024	0	CC BY 4.0

^a^ As self‐citation, we define every citing publication (excluding preprints) that is authored neither by a first nor a corresponding author of the original publication. The numbers were retrieved using OpenAlex (https://openalex.org/works).

### 4.1 Pathway Extraction

**FIGURE 3:**
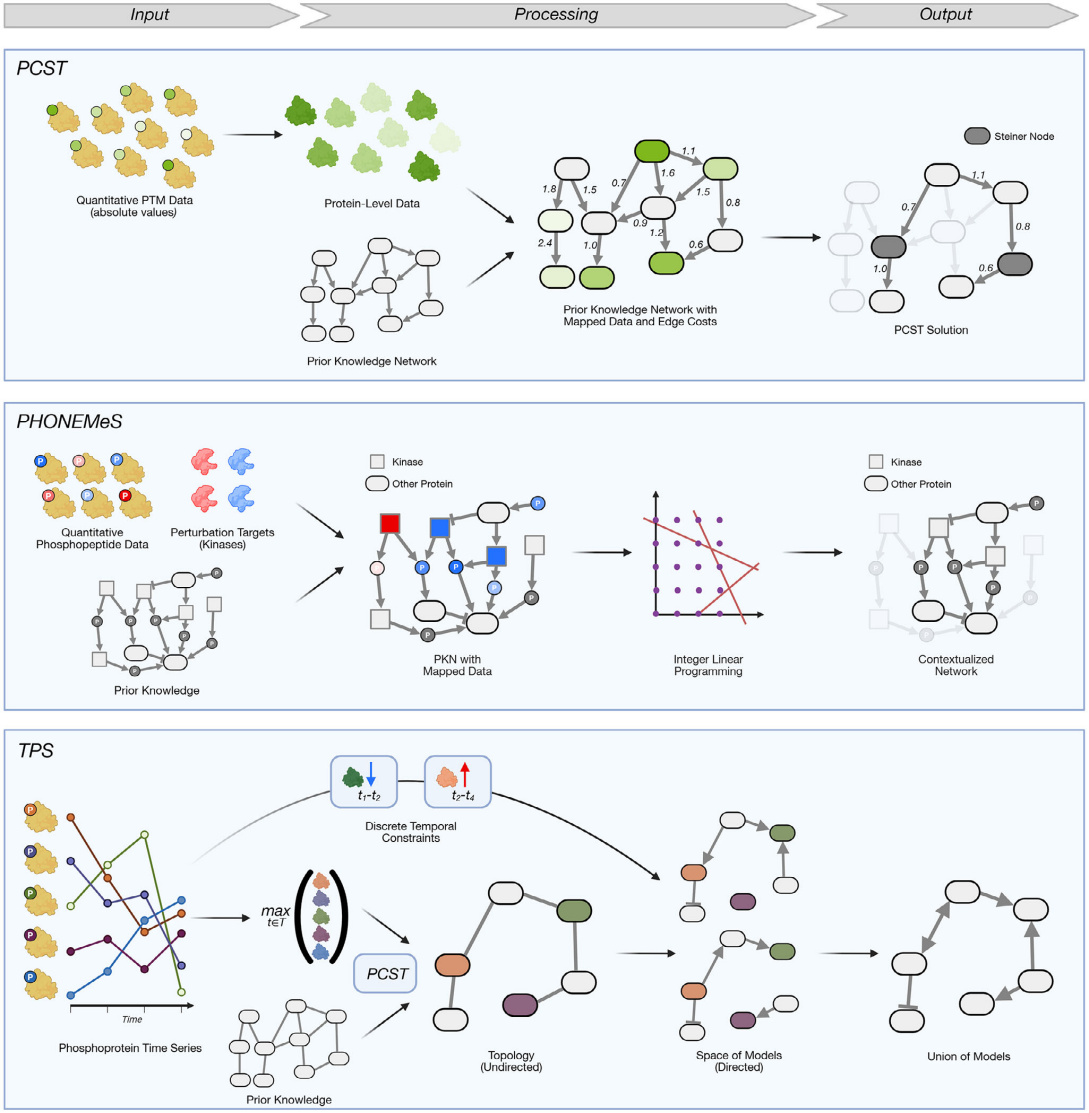
Optimization approaches for PTM network extraction. Top: The prize‐collecting Steiner tree (PCST) as implemented by Tuncbag et al. uses non‐directional quantitative data on protein level. The data are mapped to a network of prior knowledge, which can have edge costs according to the confidence in each reported interaction. The algorithm then finds a subnetwork with maximal data coverage and minimal edge costs. Additional nodes in the solution that were not part of the input data are called Steiner nodes. Middle: PHONEMeS uses phosphoproteomic data and a list of perturbed kinases and combines this with a PKN that includes kinase–phosphosite interactions. Integer linear programming is used to find a minimal subnetwork that connects targets to regulated sites. Bottom: Temporal pathway synthesizer (TPS) requires a time series of phosphoproteomic data. First, an undirected network topology is determined from the maximum fold change of each phosphoprotein using the PCST algorithm. Then, temporal constraints are compiled from the full time series data to infer a space of directed networks that are in accordance with the topology and the data. The final solution is the union of all these models.

A strategy employed by multiple extraction tools is formulating the problem as a prize‐collecting Steiner tree (PCST) or forest (PCSF), an idea pioneered by the labs of Riccardo Zecchina and Ernest Fraenkel [72, 73, 74, 75, 76] (Figure 3, Top Panel). Briefly, the nodes in the PKN are assigned prizes based on the experimental data (giving higher prizes to those that are considered more essential, for example, derived from their significance or fold change). The edges are assigned weights corresponding to the confidence of each interaction (giving higher weights to uncertain interactions). A PCST algorithm then tries to find an optimal subgraph that connects as many high‐prized nodes as possible while using only edges with minimal weight. Often, the optimal solutions include nodes that exist in the prior knowledge but do not have prizes (i.e., experimental evidence) themselves. These so‐called “Steiner nodes” correspond to pathway members that are not present (or not significant) in the data but are predicted to be important and therefore can generate hypotheses for follow‐up studies. A PCSF algorithm is similar but can predict a set of disjoint graphs (a forest) instead of a single tree, using an artificial source node to which all other nodes are connected (which effectively converts the forest into a tree again). Tuncbag et al. demonstrated that this approach can reconstruct kinase/phosphatase signaling networks [64] and later integrated it into the software OmicsIntegrator [77]. It was developed for and tested on protein‐level data only, but if data and PKN are provided on the PTM level, this would also work. Also, in its original form, the algorithm does not consider the direction of regulation (up or down), only the absolute extent. In contrast, PHONEMeS was developed for phosphoproteomics datasets and takes both the direction and quantitative values of the data into account [65]. It aims to connect user‐defined perturbation targets (i.e., the kinases activated or inactivated by the experimental stimulus) to the phosphopeptides with significant regulation (Figure 3, Middle Panel). To achieve this, PHONEMeS uses an optimization approach that searches for the smallest possible network of kinases and phosphorylation sites that explains the data. The method favors solutions that include as many of the observed changes as possible, while ensuring that the network respects the topology of the prior knowledge and does not conflict with the experimental results (the underlying mathematical approach is known as “integer linear programming”). The PKN, consisting of directed and signed PPIs and KSRs, is supplied by OmniPath, and if desired, it is simple for the user to only use selected resources or altogether supply their own PKN. The input perturbation targets can be determined by an initial kinase activity inference, but since the phosphoproteomics data and the prior knowledge KSRs are then used twice, this creates a certain dependency and might lead to overinterpretation of the PHONEMeS output. If possible, we recommend using independent information to obtain the perturbation targets (if the perturbation sources are kinase inhibitors, data from affinity pulldown experiments can be used [78, 79, 80]). In a recent preprint from the same lab, the integer linear program constraints of PHONEMeS were reformulated as a PCST algorithm [81], which underscores its high similarity to the approach by Tuncbag et al. A third tool from the same class of methods is temporal pathway synthesizer (TPS) [66] (Figure 3, Bottom Panel). It operates on time‐series phosphoproteomics data and additionally uses an undirected PPI network as input (in addition, the user may supply directions and signs for the relationships). The first step is to trim down the PKN to a relevant subnetwork, using the highest fold change of each phosphoprotein across all timepoints to extract the relevant interactions. The authors recommend using the PCST algorithm from Tuncbag et al. for this. Then, TPS converts the full input into discrete constraints of the form “Protein *A* is activated/inhibited between timepoints *x* and *y*.” The algorithm proceeds to come up with the set of possible models that fulfill these constraints, given the topology of the PKN, and then outputs the union of all valid models. A drawback of this method is that the quantitative phosphopeptide data is aggregated onto the protein level, implicitly assuming that more phosphorylation means higher protein activity. This is not true in general, as many phosphosites have functions different from activation. However, TPS could in theory also be used with a PTM‐level PKN, and this could remedy this shortcoming (although the authors do not discuss this option).

**FIGURE 4:**
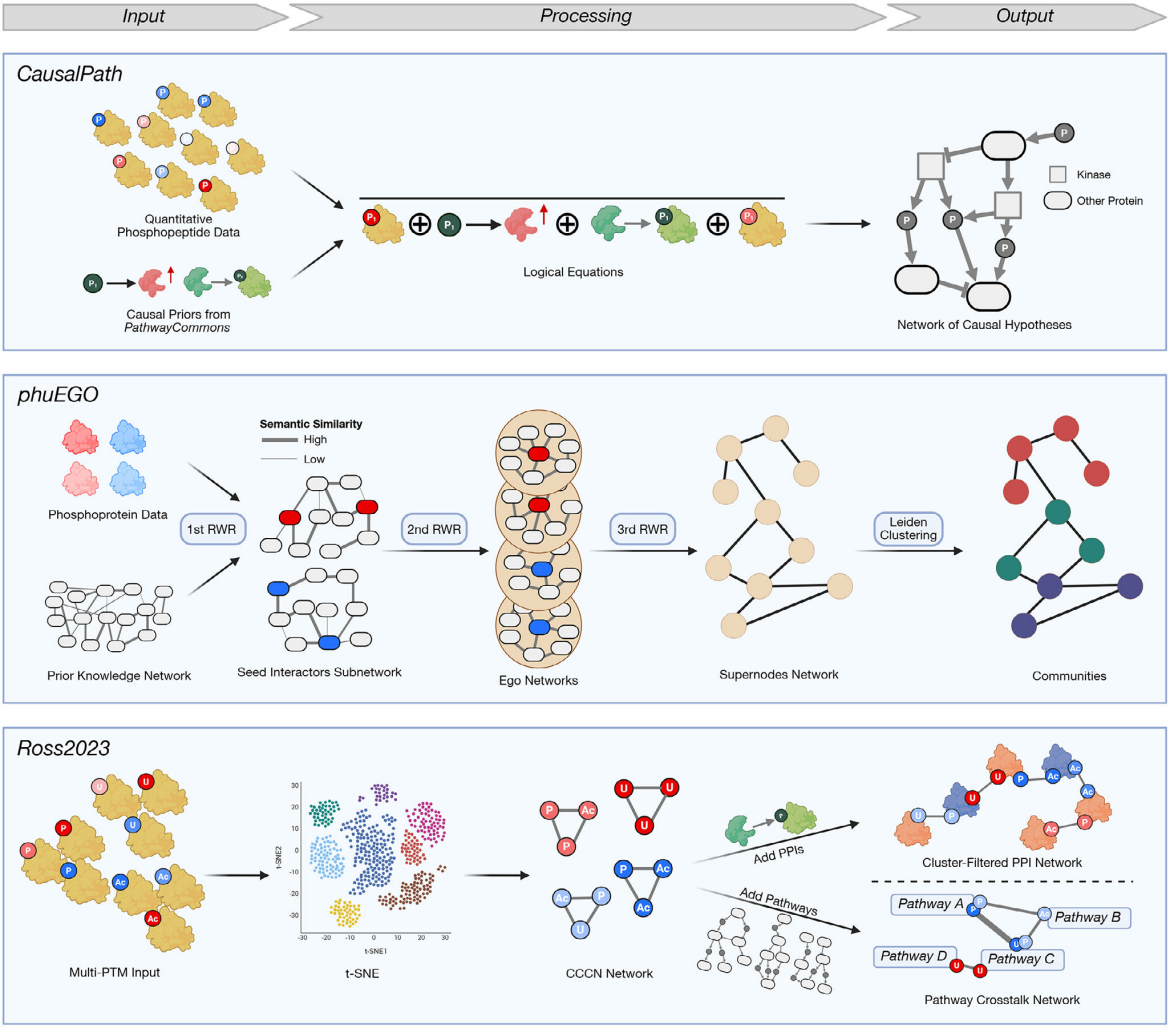
PTM pathway extraction methods beyond mathematical optimization. Top: The authors of CausalPath extracted a set of causal priors from the Pathway Commons database. User input is quantitative phosphopeptide data, which CausalPath compares to the priors by formulating logical equations. From all equations that are satisfied, a solution network is constructed. Middle: phuEGO uses protein‐level input, split into up‐ and downregulations. After applying a quantile‐based cutoff (not shown), it performs three successive iterations of random walk with restart (RWR). In the first step, up‐ and downregulated phosphoproteins are processed separately to trim down an undirected PKN into two subnetworks of proteins that interact with differentially regulated “seeds.” In the second, these are decomposed into ego networks that capture the functional neighborhood of each individual seed. In the third step, the ego networks are summarized into supernodes, and connections between them are explored to form a high‐level network. Finally, communities are detected using the Leiden algorithm. Bottom: Ross and colleagues combined multiple types of PTM from perturbation experiments. Starting with a t‐SNE on peptide‐level data, they first created a co‐cluster correlation network (CCCN), which they then combined with prior knowledge on protein–protein interactions (PPIs) to build a cluster‐filtered PPI network, and also with prior knowledge on pathways to form a network of pathway crosstalk in the context of their experiment. Ac, acetylation, P, phosphorylation; U, ubiquitination.

In contrast to these optimization‐based approaches, CausalPath is a method that focuses on utilizing the mechanistic details of the prior knowledge [67] (Figure 4, Top Panel). The authors started from the observation that pathway diagrams are heterogeneous and inconsistent. For example, a phosphorylation reaction can be modeled by a reaction from the unphosphorylated to the phosphorylated substrate, with the kinase acting as a regulator of the reaction arrow. But the kinase may also be modeled as being part of the reaction itself, or the reaction partners may only be indirectly connected via a metabolite such as ATP. Therefore, the authors defined 12 graphical patterns that are supposed to capture various ways of describing (de‐)phosphorylation and regulation of gene expression. Then they scanned the Pathway Commons pathway diagram database [82] for occurrences of these patterns, and extracted them in the form of signed directed relations. Users of CausalPath have to input phosphoproteomics data, which is discretized into binary statements of the form “Site *X* has increased/decreased phosphorylation.” Logical equations are then used to test if these statements agree with the causal priors, and those cases where the equations hold are aggregated into an output network. In contrast to TPS, CausalPath considers whether phosphosites have an activating or inactivating function for their protein. CausalPath is available as a web service or can be downloaded as a Java application, and the results can then be visualized with the Chisio BioPAX Editor [83]. The authors provided a protocol paper that describes in great detail how to prepare the input and run the software [84].

Next, there exists a group of methods that use network propagation, an approach to extracting networks by simulating the spread of information through the graph of prior knowledge. An early effort was made by the authors of TieDie [85], which was intended as a gene expression data analysis tool, but the same group demonstrated how to use the algorithm for phosphoproteomics data [86] (we refer to Garrido‐Rodriguez's review for a description of the algorithm [9]). The most recent propagation‐based method is phuEGO [68] (Figure 4, Middle Panel). phuEGO utilizes a PKN compiled from multiple PPI and KSR databases. Edge weights in the network are modeled using semantic similarity measures^2^ of the GO terms associated with each node. As user input, the tool requires two lists of protein‐level data, one for up‐, and one for downregulation (the authors recommend aggregating peptide‐level phosphoproteomic data by the maximum log fold change of all sites on the protein). A quantile‐based cutoff is then applied to obtain a set of “seed nodes.” The rest of the algorithm makes repeated use of the random walk with restart (RWR) technique: For a random walk, each edge in a graph is assigned a transition probability. Then, a “walker” is placed at one position in the graph and moves from node to node according to the transition probabilities. If the random walk is performed “with restart,” then at every step there is a certain probability that the walker returns to its start node. In PhuEGO, after determining the seed nodes, a first round of RWRs is performed from each of the seed nodes, with the combination of the PKN and the data forming the probability distribution for the edges. This is done separately for up‐ and downregulations. The results of the random walks are subnetworks that contain the most probable interactors of the seed nodes. In the second part of the algorithm, these networks are reduced using a strategy the authors term “Ego Decomposition.” For each of the seeds, a subnetwork containing the seed's local neighborhood (up to a distance of 2) is extracted. Via a second RWR run, the nodes functionally most similar to the seed are predicted (the authors suggest that they are therefore affected by the seed's level of phosphorylation). These groups of nodes are subsequently merged into so‐called “supernodes,” and the union graph of supernodes is formed. If two supernodes are connected in this union graph, a third RWR pass determines the weight of the edge between them. Finally, the supernode network is clustered into functional communities using the Leiden community detection algorithm [87].

All the algorithms described so far were developed for the analysis of phosphoproteomics data. A recent study also integrated two other types of PTM data, acetylation and ubiquitination, into a combined pathway‐centric analysis [69] (Figure 4, Bottom Panel). The authors did not publish a computational tool, but they released their code (github.com/m‐grimes/PTMs‐to‐CCCN‐and‐CFN), and their analysis strategy could be reused for other multi‐PTM studies. They treated several cell lines with different tyrosine kinase inhibitors and acquired data for each of the three PTMs separately. Then they performed t‐SNE on all measurements together and identified clusters in the PTM space. Based on this, they created three different kinds of networks. The first was on the level of PTMs, connecting those sites that show coordinated regulation. The second was on the level of proteins. To create it, the authors began from a PKN of PPIs (built from various source databases such as STRING and PSP) and removed all the edges between proteins that did not have co‐clustering PTMs. Thirdly, a pathway‐level network was created in a similar fashion, namely by starting from a pathway database (they used the currently unmaintained NCATS BioPlanet database [89]) and connecting two pathways if they contain proteins with co‐clustering PTMs. Edges between pathways were assigned a heavier weight the more co‐clustering PTMs they contained, normalized by the overall frequency of each gene across the pathway database. This multi‐level network hierarchy is an interesting approach and would be straightforward to implement with other PKNs and datasets. A potential criticism of the strategy is that only because a protein appears in a pathway, not all PTMs on it are necessarily relevant to the function it has in this pathway (with their strategy, they implicitly make this assumption).

### 4.2 Pathway Prediction

A criticism of extraction methods is that many of the true positives are, in fact, not known [81, 90, 91], so the PKNs are a very incomplete representation of reality. This makes network prediction methods an attractive field of research. To our knowledge, no method has been published that operates on large‐scale PTM data, potentially because both the implementation and evaluation of such tools are harder compared to extraction methods. Nevertheless, we found two tools that hold the potential of being extended in this direction.

**FIGURE 5:**
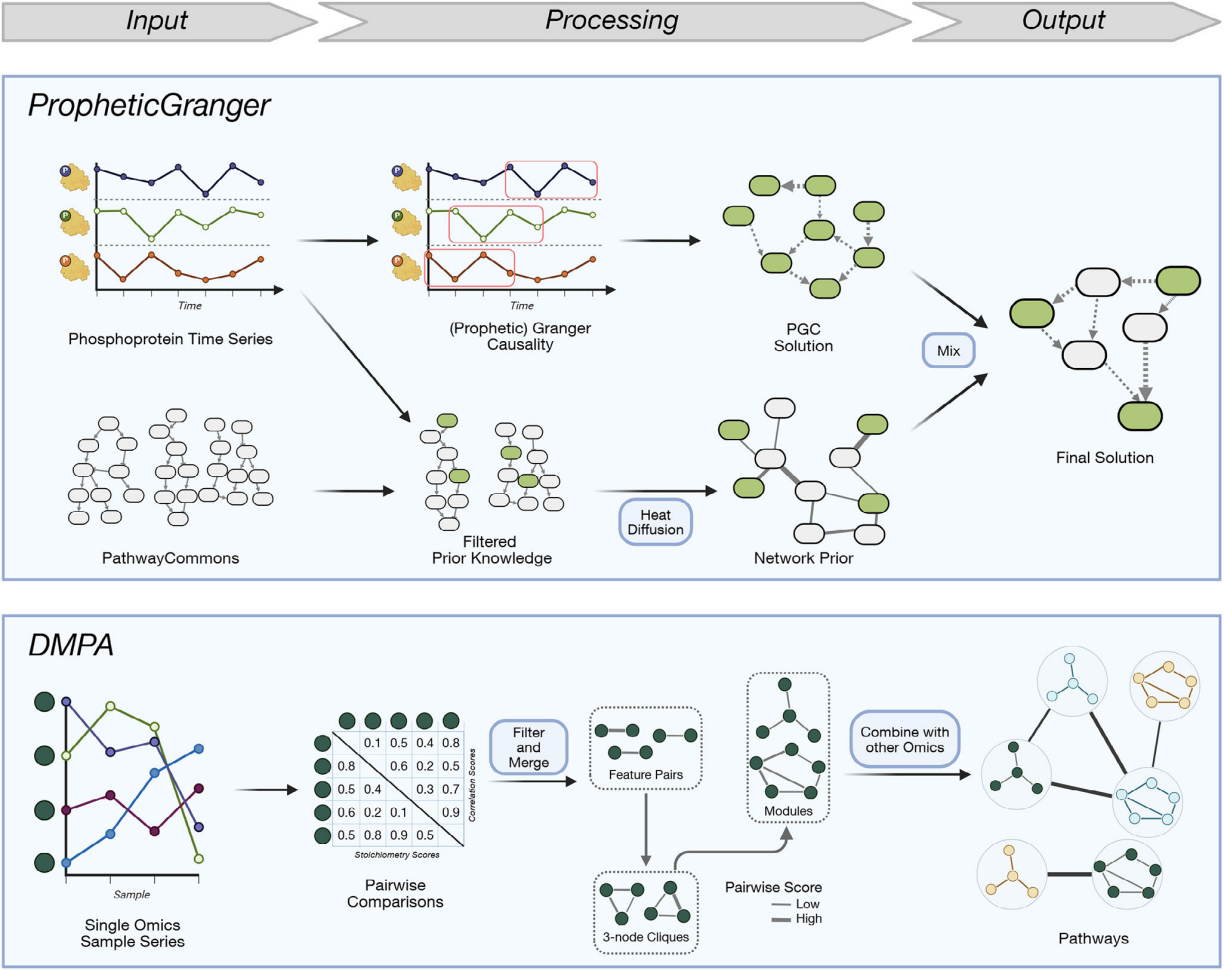
Network prediction tools. Top: Prophetic Granger requires a time series of phosphoprotein data as input and uses Granger causality to determine if the profiles of the proteins are temporally related, which de novo predicts a directed network of causal interactions. In parallel, a pathway database is filtered for networks containing the measured proteins, and heat diffusion is used to construct an undirected network prior. The two solutions are mixed to give the final (directed) solution. Bottom: DMPA needs a series of multi‐omics datasets (e.g., multiple timepoints or perturbations) and operates independently of prior knowledge. First, pairs of PTM sites from a single type of omics are identified that have a high correlation and conserved stoichiometry. In a similar fashion, these are combined into cliques of three nodes, larger modules, and finally combined with modules from other omics to form (undirected) pathways at site‐level.

In 2016, a challenge was conducted to benchmark models that infer causal signaling networks from time‐series phosphoprotein data [92]. Prophetic Granger emerged as the top‐performing method out of 74 submissions [70]. It uses the statistical concept of Granger causality to de novo determine a network of directed interactions between the measured proteins that help explain their temporal profiles (Figure 5, Top Panel). In parallel, Prophetic Granger constructs a network of prior knowledge, starting from all pathways in Pathway Commons [82] that contain the proteins of interest and unifying them into a single undirected network. This is achieved using heat diffusion, an established strategy in network analysis in which a flow of heat is simulated to draw conclusions about the topology and connectivity of a network. The result of the heat diffusion is an undirected network. The two independent networks are then averaged to produce the final (directed) output, which was shown to perform better in reconstructing known signaling networks than either of the individual methods (improving the AUROC from 0.55 to 0.797). However, the authors state that the top results were achieved when combining prior knowledge and prophetic Granger inference in an 80/20 ratio. Also, the underlying data came from reverse‐phase protein lysate arrays, so only a selection of 45 phosphorylation sites was profiled. The method would need adjustments in order to be applicable to global PTM data (for instance, how to select the pathways from which to form the PKN), but in principle the approach is interesting.

The second method, DMPA, works on an arbitrary combination of multi‐omics and entirely without prior knowledge [71] (Figure 5, Bottom Panel). It follows a three‐step procedure of inferring signaling networks: First, features (i.e., individual quantitative profiles on PTM site level) are compared within each type of omics, and two types of pairwise scores are computed:

- A Spearman rank‐correlation score that quantifies how well two sites are correlated with each other across the samples of one omics type.
- A stoichiometry score that assesses how conserved the ratio between the two features is (which can indicate an interaction).

The feature pairs are then filtered using cutoffs. Second, the feature pairs are compared to first create 3‐node cliques and then modules. Third, the modules are combined into pathways, again using a pairwise comparison‐and‐merging strategy. At this step, the different omics layers are also merged. The authors evaluated this approach against ground truths from STRING and PSP and observed better performance than earlier approaches in the field. The MATLAB‐based tool has been published as an executable Windows desktop application with a graphical user interface, making it straightforward to use for non‐bioinformaticians. The authors only evaluated it on a combination of phosphoproteomics, interactomics, transcriptomics, and proteomics data, but it is, in principle, also applicable to multiple PTM datasets of the same biological system. Being a purely data‐driven tool can be an advantage, but users of DMPA also need to keep in mind that the tool can only infer correlations, not causalities (which is why the resulting networks are undirected).

## 5 Integrative Analysis Platforms

Besides individual tools for enrichment or pathway reconstruction, a number of applications have been developed that incorporate multiple functionalities for more comprehensive analysis of user datasets. These often integrate some of the methods described in the previous sections. The benefit of such integrations is that they make the tools simpler to use. Especially users without a background in bioinformatics or researchers from other fields would not use those individual tools in the first place. On the other hand, for the sake of being intuitive, these platforms may take away some of the customizability that the individual tools offer, for example, by fixing a parameter to a value that works in most cases or not specifying in detail the underlying algorithms. As for the previously covered tools, there is a bias on phosphorylation versus other PTMs. A summarizing overview is provided in Table 3.

**TABLE 3:** Integrative platforms for analyzing PTM data with a pathway focus.

Name	URL	Focus	Latest release	License
Phosphomatics [93]	https://phosphomatics.com	Connect phosphoproteomics data with prior knowledge on KSRs; implements KSEA	August 2023	Custom (https://phosphomatics.com/license)
PhosFate [94]	http://phosfate.com/	Compare phosphoproteomics data with the Kinase Regulation Atlas	February 2016	CC BY‐ND 3.0
WebGestalt [95]	https://www.webgestalt.org	Multi‐omics enrichment analysis	May 2024	Free
PhosR [96]	https://pyanglab.github.io/PhosR/	R package for preprocessing, enrichment analysis, and kinase–substrate prediction of phosphoproteomics data	May 2025	GPL 3.0
Selphi2 [97]	https://selphi2.com/	Prediction of upstream kinases using a machine learning model trained on PhosphoSitePlus; kinase activity and pathway enrichment analyses; network reconstruction with PCSF	May 2025	MIT
PTMNavigator [98]	https://www.proteomicsdb.org/analytics/ptmNavigator https://github.com/kusterlab/enrichment‐server	Visual projection of PTM onto pathway diagrams; creation of custom diagrams Standardized pathway enrichment and kinase activity inference	January 2025	Apache 2.0
Cytoscape [99, 100]	https://cytoscape.org	General‐purpose desktop application for biological network visualization and analysis	October 2024 (Desktop Version) June 2025 (Web Version)	Desktop: LGPL 2.1 Web: MIT

For example, Phosphomatics is a suite of tools for downstream analysis of phosphoproteomics data [93]. Uploaded datasets are annotated with upstream kinases, pathways, and other contextual information. Users can conduct analyses such as Kinase‐Substrate Enrichment Analysis (KSEA; using the implementation by Wiredja et al. [101]) or pathway enrichment (method not specified, likely a Fisher test) and visualize dynamically generated kinase–substrate networks. The documentation also mentions a pathway‐centric view, where data could be mapped onto static KEGG pathway diagrams, but this functionality seems to be no longer available. Another phosphorylation‐centric website is PhosFate [94]. It offers users the opportunity to explore a large collection of perturbation studies and inferred kinase activities. Custom data can be uploaded and compared to this atlas. Users seeking to perform enrichment analyses, particularly on multi‐omics datasets, are referred to WebGestalt [95] and PhosR [96]. Established in 2005, WebGestalt recently integrated PTMSigDB into its arsenal of databases and supports all three classes of pathway enrichment analyses (for topology‐based enrichments, an RWR approach is used). PhosR, in contrast, is an R package that performs both processing and analysis of phosphoproteomics data. The authors pre‐analyzed a panel of phosphoproteomic experiments and identified a set of residues with little variance in phosphorylation levels across conditions, which they termed stably phosphorylated sites. When processing user data, these sites are used to correct for batch effects. Another concept introduced by the authors is that of “signalomes,” which describes the entirety of sites and proteins that are controlled by the signaling of one kinase. PhosR combines this concept with both site‐ and gene‐level ORA and FCS enrichment algorithms to allow multidimensional analysis of user data. It also includes a method for kinase–substrate inference that is based on the AdaSampling algorithm [102]. Next, there is SELPHI_2.0_, a web platform with many possible analysis options for phosphoproteomics data [97]. At its core is a network of kinase–substrate predictions, which the authors created using a random forest classifier that was trained on PSP annotations and a set of features that included, among others, the functional scores from the Beltrao lab [103] and the in vitro KSRs from the Ishihama lab [35]. Researchers can make use of this network to predict candidate kinases for their phosphorylation data and to predict kinase activities (using GSEA as the enrichment algorithm). Furthermore, the web platform offers ORA enrichment with Fisher or hypergeometric tests against multiple databases, including KEGG pathways and PTMSigDB signatures, and users can extract signaling networks from their data using an implementation of the PCSF algorithm [104].

A software that is not limited to phosphorylation is PTMNavigator, which was recently developed by the authors of this review and is available on the ProteomicsDB platform [98]. Its central functionality is the overlaying of pathway diagrams with user PTM perturbation data, which enables users to trace the flow of signal across biological networks. Users can either work with canonical pathways (KEGG, WikiPathways) or create custom graphs. The second pillar of the project is a server that provides standardized access to a panel of pathway enrichment and kinase activity inference algorithms. Datasets uploaded to PTMNavigator are automatically sent to this service, and the results are used to sort pathways by putative relevance, highlight differentially active kinases in the diagrams, and create data‐driven kinase signaling networks (making use of the PHONEMeS algorithm described above). The enrichment server can also be directly queried via an API; instructions and examples for this are available at https://github.com/kusterlab/enrichment‐server.

Lastly, we would like to mention Cytoscape, which is a generic software for visualization and analysis of biological networks [99]. Many of the tools we have described here either incorporate Cytoscape in their workflow or produce output that can be imported into Cytoscape. It supports many of the widely accepted formats for network representation, including SIF, GML, and SBML. In 2025, a web instance was released, which simplifies the usage compared to the desktop version and also presents an incentive for collaborative research [100].

## 6 Discussion and Outlook

PTM data often are not consistent at the individual site level across different datasets, yet tend to converge when analyzed from a pathway‐level perspective [81]. This discrepancy underscores the value of higher‐level analyses: pathway‐centric approaches help us “see the forest for the trees.” The emergence of the herein presented methods for pathway‐centric PTM research marks a crucial step forward in extracting functionally relevant insights from noisy and heterogeneous datasets. In the following, we discuss what we consider to be the most pressing issues in this research area and what future developments can be expected.

### 6.1 On the Lack of Functional Annotations

A fundamental limitation for all prior‐knowledge‐based methods lies in the annotations. Enrichment analyses are often performed at the gene or protein level—not because this is biologically ideal, but because annotations for genes and their products are far more comprehensive and standardized than those for PTMs. Many pathways are not, or only sparsely, covered by PTM‐level annotations. Most biological pathway databases are gene‐ or protein‐centric, even though it has been acknowledged that many pathways would more accurately be modeled on the PTM level. The most comprehensive resource in this regard is PTMSigDB, but its coverage remains limited. Also, while PTMSigDB, at the time of writing, included 11 types of modifications, 99.6% of the annotations are for phosphorylation sites. An extension to this would be very desirable. Similarly, Ochoa et al.’s functional PTM scores are a well‐appreciated support for researchers but remain confined to phosphosites [103]. Only some of the currently detectable PTM sites appear to influence protein function, localization, or interactions [105, 106]. This might lead to spurious edges when using them for predicting networks, which makes estimators of functionality attractive to limit false positives. Moreover, a lot of functional annotations turn out to be incorrect when subjected to validation experiments, so researchers should keep in mind that not every entry has the same level of confidence, and annotations may change as new evidence is obtained [107, 108, 109]. It is important that updates to annotation resources are promptly integrated into the tools that use them and to diligently report which versions were used when publishing an analysis [110].

### 6.2 On the Limitations of Enrichment Tools

It is tempting to take the results of pathway enrichment analyses at face value. However, we must remain aware of the assumptions that underlie these methods. Pathway enrichment often treats pathways as isolated and independent units, which is an artificial simplification imposed for computational tractability. In reality, biological pathways are deeply interconnected, with considerable crosstalk and redundancy. We generally agree that pathway enrichment methods are useful as hypothesis‐generating tools. However, we caution against the common practice of presenting them as definitive endpoints of a study, often without any experimental follow‐up. This practice limits their scientific utility. Instead, such analyses should serve as the starting point for follow‐up experiments that validate and refine the inferred hypotheses.

### 6.3 On the Disparity Between Mathematical Models and Biological Reality

Many of the mathematical frameworks used in PTM pathway analysis impose constraints that are not in line with the true nature of the studied system. Distance‐based methods like PCST assume that the most informative or biologically relevant connections are the shortest paths. While computationally convenient, this assumption is often too simplistic. Another common constraint is the requirement that inferred networks be acyclic; this is, for example, the case in all PCST‐based algorithms. In reality, feedback loops and cycles are prevalent in signaling networks. Supervised learning classifiers also face difficulties. A major issue here is the identification of true negatives. Given the abundance of false negatives in interaction data, it is misleading to assume that randomly selected pairs of proteins or PTMs do not interact. Some studies, such as PhosFormer [111] and a recent knowledge‐graph‐based edge prediction approach for PKNs by Gavali and colleagues [112], have attempted more sophisticated approaches to selecting negatives, but these efforts remain limited to kinase–substrate relationship predictions. For pathway inference, constructing an appropriate negative ground truth remains an unsolved challenge, and we urge researchers to carefully evaluate how a given tool handles this issue.

### 6.4 On the Importance of Benchmarks

Compared to transcriptomics, proteomics—and in particular PTM‐centric proteomics—still lacks mature, standardized methods for pathway‐level analysis. While the development of new tools can be valuable, we often observe the field reinventing the wheel rather than refining what already exists. A major obstacle is the lack of comparability across tools. Differences in input data formats, preprocessing steps (e.g., raw intensities vs. *z*‐scores vs. *p* values), handling of peptide‐to‐site mappings, assumptions about localization ambiguity, and statistical frameworks make it difficult to meaningfully compare results. Moreover, the absence of well‐defined ground truths further complicates validation. Many studies frame their methods as superior by constructing use cases in which their tools perform best. However, these evaluations are often biased, and for most readers—especially those without a computational background—it is difficult to discern whether the benchmarks are fair or artificially favorable. As noted by Nayar et al., the lack of standardized benchmarking practices in the field continues to impede progress [91]. Benchmarking is crucial for the fair and reproducible evaluation of new methods. We previously referenced the kinase activity inference benchmark by Müller‐Dott, which provides a framework for evaluating both algorithms and KSR resources [8]. In pathway reconstruction, however, there are currently not enough comparable initiatives. The HPN‐DREAM challenge (last held in 2016) served as a valuable platform, and we believe that a new edition would greatly benefit the community. More recently (in 2023), Sriraja et al. performed their own benchmark but restricted their evaluation to methods not using PKNs [113]. We emphasize that independent benchmarks reduce the risk of biased self‐evaluation, be they intentional or not. In our view, the most important criterion for methods using pathway databases is robustness. For example, the MAPK pathway differs in detail between databases such as KEGG and WikiPathways, yet a good inference algorithm should produce consistent conclusions regardless of which version is used. If conclusions change depending on the database, the method is probably unreliable. Benchmarks should be designed in a way that accounts for this kind of variability.

## 7 Concluding Remarks

In the last two decades, the study of PTMs in the context of pathways has seen significant advancements. A plethora of computational methods have been released that attempt to make experimental results tangible. We have reviewed a representative selection of these methods and pointed out the respective strengths and shortcomings. In our opinion, the focus of the field needs to shift towards the development of fair and widely applicable benchmarks so that researchers have a transparent and traceable groundwork to make a rational choice of which method to use for which scientific question. We note that coming up with ground truths is a very difficult problem, which seems to be why the development of novel tools is usually preferred over such benchmark studies. On the experimental side, we endorse unbiased and global efforts such as the Kinase Library [20, 21]. Projects such as this are laying the foundation for improving the quality and coverage of functional PTM annotations and should be prioritized over studying only well‐known proteins and interactions. To achieve these goals, a close cooperation between computational and experimental scientists will be quintessential.

## Conflicts of Interest

B.K. is a cofounder and shareholder of MSAID. He has no operational role in the company. The other authors declare that they have no competing interests.

## Data Availability

The authors have nothing to report.

## Footnotes

1 We note that the terminology used in the field is not consistent. The term Gene Set Enrichment Analysis (GSEA) is sometimes used to summarize both ORA and FCS, in order to separate them from PT methods. Sometimes it represents FCS only, and sometimes it refers to the individual method developed by Subramanian and colleagues [52]. We will stick to this last definition to avoid misunderstandings.

2 Semantic similarity measures are metrics that assess how similar text segments are in meaning. phuEGO uses simGIC, which has been shown to perform well for the semantic comparison of GO terms [88]
